# Antigenic Dark Matter: Unexplored Post-Translational Modifications of Tumor-Associated and Tumor-Specific Antigens in Pancreatic Cancer

**DOI:** 10.3390/cancers17213506

**Published:** 2025-10-30

**Authors:** Amin Safa, Idris Vruzhaj, Marta Gambirasi, Giuseppe Toffoli

**Affiliations:** 1 Institute of Research and Development, Duy Tan University, Da Nang 550000, Vietnam; 2 School of Medicine and Pharmacy, Duy Tan University, Da Nang 550000, Vietnam; 3 Experimental and Clinical Pharmacology Unit, Centro di Riferimento Oncologico di Aviano (CRO) IRCCS, 33081 Aviano, Italy; idris.vruzhaj@gmail.com (I.V.); marta.gambirasi@cro.it (M.G.); 4 Ludwig Institute for Cancer Research, Department of Oncology, University of Lausanne and Lausanne University Hospital (CHUV), 1011 Lausanne, Switzerland; 5 Department of Pharmaceutical and Pharmacological Sciences, University of Padua, 35131 Padua, Italy; 6 Department of Immunology, School of Medicine, Zabol University of Medical Sciences, Zabol 98616-15881, Iran; 7 Department of Life Sciences, University of Trieste, 34127 Trieste, Italy

**Keywords:** pancreatic ductal adenocarcinoma, post-translational modifications, immune Evasion, immunopeptidomics, glycosylation, citrullination

## Abstract

**Simple Summary:**

Pancreatic ductal adenocarcinoma is very hard to treat, and most patients do not benefit from current immune-based drugs. One reason may be that we are looking for the wrong targets. Proteins in cancer cells often undergo small chemical changes after they are made. These changes (PTMs) can create new “flags” that the immune system can see, but usual tools do not catch them. We describe a clear plan to find these PTM targets with modern lab methods, check them in patient-derived models, and connect them to tests and treatments like vaccines or engineered T cells. This could make immunotherapy work for more people with PDAC.

**Abstract:**

**Background:** Pancreatic ductal adenocarcinoma (PDAC) exhibits marked resistance to immunotherapy. Beyond its characteristically low tumor mutational burden, post-translational modifications (PTMs) remodel the immunopeptidome and promote immune escape through reversible, enzyme-driven programs. **Subject Matter:** We synthesize evidence that aberrant glycosylation, O-GlcNAcylation, phosphorylation, and citrullination constitute core determinants of antigen visibility operating within spatially discrete tumor niches and a desmoplastic stroma. In hypoxic regions, HIF-linked hexosamine metabolism and OGT activity stabilize immune checkpoints and attenuate antigen processing; at tumor margins, sialylated mucins engage inhibitory Siglec receptors on innate and adaptive lymphocytes; within the stroma, PAD4-dependent NET formation enforces T cell exclusion. We also delineate technical barriers to discovering PTM antigens labile chemistry, low stoichiometry, and method-embedded biases and outline practical solutions: ETD/EThcD/AI-ETD fragmentation, PTM-aware database searching and machine-learning models, and autologous validation in patient-derived organoid–T cell co-cultures. Finally, we highlight therapeutic strategies that either immunize against PTM neoepitopes or inhibit PTM machinery (e.g., PAD4, OGT, ST6GAL1), with stromal remodeling as an enabling adjunct. **Conclusions:** PTM biology, spatial omics, and patient sample models can uncover targetable niches and speed up PDAC vaccination, TCR, and enzyme-directed treatment development.

## 1. Introduction

### 1.1. Pancreatic Cancer: Immune Evasion Beyond Mutational Load

Pancreatic ductal adenocarcinoma (PDAC) remains exceptionally challenging to treat; even with contemporary multimodal regimens, the five-year survival rate is below 12% [1]. This grim outlook largely reflects presentation at advanced stages and robust tumor-intrinsic immune evasion [2]. Although recurrent oncogenic lesions, most notably KRAS substitutions at G12 (G12D/V/R) in nearly 90% of cases [3] and TP53 (identified in about 77%) [4], represent prototypical tumor-specific antigens (TSAs), efforts to therapeutically target them have achieved limited success. Clinical studies of KRAS-directed mRNA vaccines (e.g., mRNA-5671; NCT03948763) and TP53-focused synthetic long-peptide vaccines have failed to produce objective responses in more than 90% of recipients, underscoring the constraints of mutation-centric immunotherapies in PDAC [5,6].

**Figure 1:**
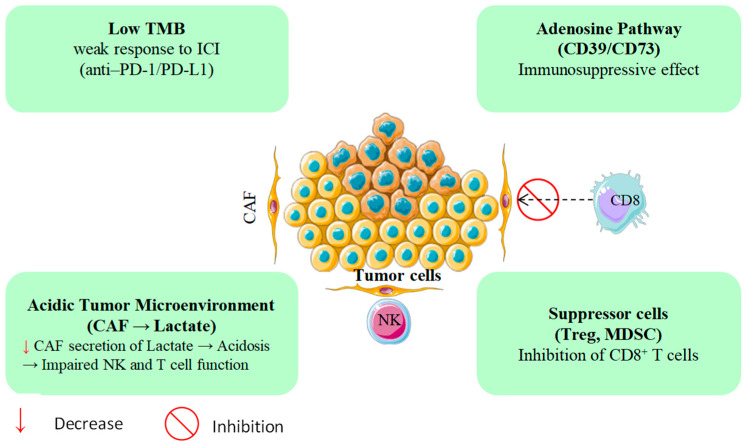
PDAC’s immune evasion mechanisms beyond low TMB.

The pronounced immunoresistance of PDAC is largely driven by its dense, desmoplastic, and immunosuppressive tumor microenvironment (TME) [7,8]. Stromal components, particularly cancer-associated fibroblasts (CAFs), can constitute up to 80% of the tumor mass and secrete immunomodulators such as TGF-β, CXCL12, and IL-10 [9]. Collectively, these mediators impede the infiltration and effector function of cytotoxic CD8^+^ T cells while promoting the expansion of immunosuppressive subsets, including FOXP3^+^ regulatory T cells and CD11b^+^Gr1^+^ myeloid-derived suppressor cells [10]. These immune evasion mechanisms, beyond just low TMB, are depicted in Figure 1 Compounding this, PDAC typically exhibits one of the lowest tumor mutational burdens among solid tumors, often fewer than one nonsynonymous mutation per megabase which correlates with poor responsiveness to immune checkpoint inhibitors [11,12]. Accordingly, anti-PD-1/PD-L1 monotherapy yields consistent response rates of under 5%, and phase III trials have not demonstrated durable therapeutic benefit in PDAC patients [13,14,15,16,17]. Recent studies have highlighted the essential function of PTMs in the immunopeptidome of PDAC. Ely et al. demonstrated that PDAC tumors possess concealed antigens resulting from PTMs that are undetectable by conventional genomic assays. PTM-derived antigens may serve as targets for immunotherapy, a promising approach for treating PDAC patients with limited mutations [18].

Beyond its structural barriers, PDAC exhibits profound metabolic dysregulation that further entrenches immune escape [19]. Tumor hypoxia drives extracellular adenosine accumulation via the CD39/CD73 ectonucleotidase axis, suppressing dendritic cell maturation and compromising T cell priming [20,21,22]. In parallel, the heightened glycolytic flux of CAFs generates excess lactate, acidifying the tumor milieu, and blunting natural killer (NK) cell cytotoxicity [23]. In keeping with these constraints, recent trials of PDAC immunotherapy show limited activity: no complete responses to TSA-directed vaccines or checkpoint inhibitors and a median progression-free survival under 2.5 months, a pattern largely attributable to an immunosuppressive microenvironment enriched for myeloid-derived suppressor cells and CAFs (Table 1). Taken together, these data underscore the need for innovative therapeutic strategies [24,25].

Collectively, these observations argue for a reorientation of antigen discovery away from an exclusive focus on mutational neoantigens and toward non-mutational targets, particularly PTMs such as glycosylation, phosphorylation, and citrullination. These alterations are frequent in PDAC and may be recognized through non-canonical, MHC-independent pathways, potentially circumventing both stromal barriers and the immunologic impediments that limit conventional immunotherapies.

### 1.2. Post-Translational Modifications: The Subsequent Frontier in Antigen Discovery

PTMs including glycosylation, phosphorylation, citrullination, and ubiquitination govern protein function and reconfigure the antigenic landscape of malignant cells [34]. Unlike DNA mutations, PTMs generate neoantigenic determinants by chemically altering self-proteins, thereby providing a non-mutational route to immune recognition [35]. PDAC; such modifications both facilitate immune escape and present actionable targets for therapy [36].

Aberrant glycosylation, particularly of mucins such as MUC1, drives the expression of tumor-associated carbohydrate antigens (e.g., Tn and sialyl-Tn [STn]) [37]. These glycans can sterically shield immunodominant peptide epitopes, diminishing detection by cytotoxic CD8^+^ T cells [38]. Immunopeptidomic analyses using mass spectrometry on patient-derived xenografts substantiate this masking effect in PDAC [39,40]. Conversely, citrullination catalyzed by peptidylarginine deiminases (PAD2 and PAD4) can create immunogenic neoepitopes exemplified by citrullinated vimentin that are efficiently presented on MHCI and recognized by autoreactive T cells, even in tumors with very low mutational burdens [41,42].

**Figure 2:**
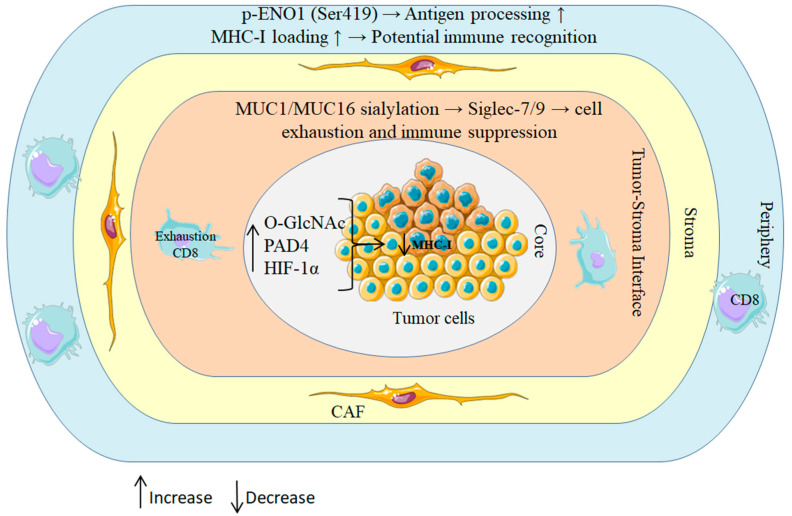
Spatial regulation of PTMs in PDAC shapes immune outcomes.

Spatial proteomics further indicates that PTMs are patterned rather than randomly dispersed within the tumor microenvironment: hypoxic niches exhibit elevated PAD4 and O-GlcNAc transferase (OGT) activity, forming localized PTM “hotspots” that coincide with immune-exclusion zones in PDAC (Figure 2) [43,44,45]. These distributions have functional consequences. Phosphorylation of tumor antigens, for instance, ENO1 at serine 419, can enhance proteasomal processing and promote efficient MHC-I loading, whereas O-GlcNAcylation of components of the antigen-processing machinery impairs peptide presentation [46,47]. Reflecting this context dependence, sialylated MUC16 dampens immunity through the engagement of inhibitory Siglec receptors on natural killer (NK) cells, while phosphorylated HER2-derived peptides elicit robust T cell responses in breast and pancreatic cancers [48,49].

Clinical validation of PTM-derived targets is gaining momentum. In preclinical models, a chemically defined synthetic MUC1 glycopeptide vaccine directed at tumor-associated epitopes such as sTn elicited strong Th1-biased responses and activated cytotoxic T cells in murine PDAC [50]. Nevertheless, substantial hurdles remain: many PTM-modified peptides are scarce and short-lived, rendering them difficult to detect with conventional mass spectrometry [51,52,53]. Moreover, distinguishing biologically consequential (“driver”) PTM events such as PAD4-mediated histone citrullination linked to neutrophil extracellular trap formation from incidental changes requires single-cell PTM-omics with high spatial and temporal resolution [54,55].

Overcoming these barriers could open a new class of immunotherapies for antigenically “cold” tumors like PDAC. PTM-focused interventions may enable next-generation vaccines, T cell receptor-engineered cellular therapies, and selective small-molecule agents, for example, PAD4 inhibitors, to disrupt citrullination-driven immunosuppression. As the PTM-centered immunobiology of PDAC is mapped with greater precision, these alterations are poised to become integral design parameters for tailored immunotherapeutic strategies.

This review focusses on four PTM axes (glycosylation, O-GlcNAcylation, phosphorylation, citrullination) to provide depth in accordance with PDAC evidence; additional PTM programs (e.g., SUMOylation, ubiquitination, acetylation, β-hydroxybutyrylation, methylation, ferroptosis) are summarized in Table 2.

Schematic of concentric tumor zones illustrating how patterned PTMs rewire antigen processing and immune surveillance. Hypoxic core: HIF-1α–driven OGT activity elevates O-GlcNAcylation and PAD4-mediated citrullination, jointly reducing MHC-I peptide display. Tumor stroma interface: aberrant MUC1/MUC16 sialylation ligates inhibitory Siglec-7/9 on T and NK cells, promoting functional exhaustion; a CAF-rich stroma further enforces exclusion. Peripheral rim: context-dependent ENO1 phosphorylation (Ser419) enhances antigen processing and MHC-I loading, enabling CD8^+^ T cell recognition. Collectively, these spatial PTM programs create immunosuppressive niches centrally while permitting immune detection at the margins.

## 2. The Hypothesis of Antigenic Dark Matter

Antigenic black matter refers to the PTM-rich part of the PDAC immunopeptidome that is undoubtedly quite large but is often missed since acquisition settings, database searches, and immune-tolerance filters all down-weight fragile, low-abundance PTMs. Data from PDAC and other malignancies indicate that glycosylated, O-GlcNAcylated, phosphorylated, and citrullinated ligands facilitate immune evasion, whereas PTM-aware mass spectrometry, incorporating ETD/EThcD, DIA, multi-engine rescoring, and machine-learning inference is progressively enhancing detection capabilities. This “hidden” layer thus presents a promising reservoir of targets for vaccines, T cell receptors, and enzyme- or pathway-directed treatments in PDAC.

**Figure 3:**
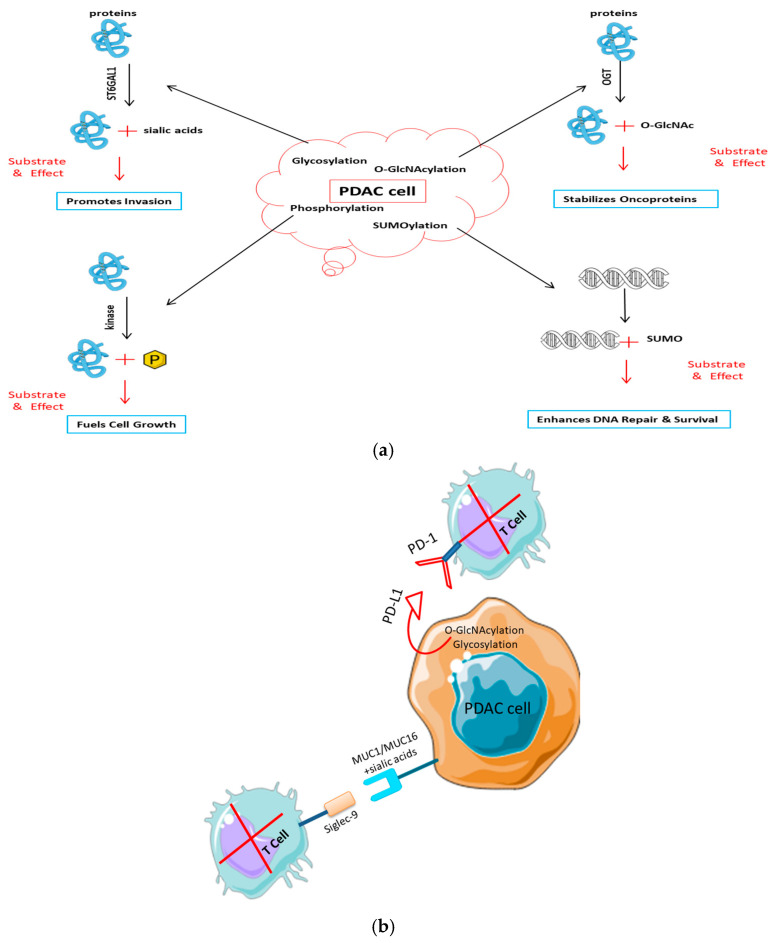
Post-translational modifications drive pancreatic cancer progression and immune evasion. (**a**) PTMs in tumor development and signaling. Activated key PTM pathways in PDAC cells lead to cancer growth: glycosylation (e.g., by ST6GAL1) of receptors like EGFR promotes invasion; O-GlcNAcylation by OGT stabilizes oncoproteins such as β-catenin to drive proliferation; and kinase-mediated phosphorylation (e.g., MAPK/ERK) drives uncontrolled growth. (**b**) PTMs in avoiding the immune system. PTMs change the surface of tumor cells to make them less immune: Sialylated mucins (MUC1/MUC16) bind to inhibitory Siglec receptors on T and NK cells. O-GlcNAcylation and glycosylation stabilize the PD-L1 checkpoint protein, which stops T cells from entering.

The “antigenic dark matter” hypothesis holds that PTMs constitute a vast, underexplored reservoir of tumor antigens that occupy a substantial share of the immunopeptidome [69] but largely escape detection because of methodological blind spots and layers of immunological tolerance (Figure 3a). Unlike mutation-derived neoantigens, PTM-bearing peptides arise through dynamic, enzyme-mediated processes and can markedly expand the antigenic diversity of cancers [35]. In PDAC, mass spectrometry-based immunopeptidomics indicates that a considerable proportion of MHC class I-presented peptides carry PTMs, broadening the antigenic repertoire and yielding putatively tumor-specific targets with clear relevance for precision immunotherapy [36,70]. These observations emphasize that PTMs can modulate tumor immunogenicity independently of TMB.

### 2.1. Mechanistic Bases of PTM-Mediated Immune Evasion

PTMs promote immune escape in PDAC through multiple antigen-focused mechanisms:

- Glycosylation: Aberrant hypersialylation of mucins (e.g., MUC1, MUC16) generates a dense glycan shield that interferes with T cell receptor engagement, while simultaneously triggering inhibitory Siglec receptors on NK cells to dampen cytotoxicity [36,71,72].
- Citrullination: PAD4-driven conversion of arginine to citrulline in proteins such as vimentin and histones can create neoepitopes; however, central tolerance particularly thymic deletion of autoreactive clones limits effective recognition [73,74].
- Phosphorylation: Hypoxia-associated phosphorylation of metabolic enzymes (e.g., ENO1 S419) enhances peptide processing and MHC-I display [46]. Paradoxically, increased antigen presentation may recruit CD73^+^ regulatory T cells via adenosine signaling [75], establishing localized immune tolerance.

### 2.2. Neoantigen Diversity Beyond the Genome

In contrast to fixed genetic alterations, PTMs provide a reversible and time-sensitive layer of antigenic plasticity [76]. This metabolic agility enables tumors to rapidly recalibrate their antigenic surface under immune selection [77,78].

- Dynamic Glycosylation: Real-time modulation of sialylation by enzymes such as ST6GAL1 can reconfigure immune visibility within hours [79,80].
- Contextual Immunogenicity: Although many PTMs favor immune evasion, some enhance recognition. Notably, citrullinated peptides derived from oncogenic KRAS (G12D) elicit CD8^+^ T cell responses in PDAC patients who do not respond to the corresponding unmodified peptides, revealing fresh avenues for neoantigen targeting [33,81,82].

### 2.3. Technical Barriers and Emerging Solutions

**Figure 4:**
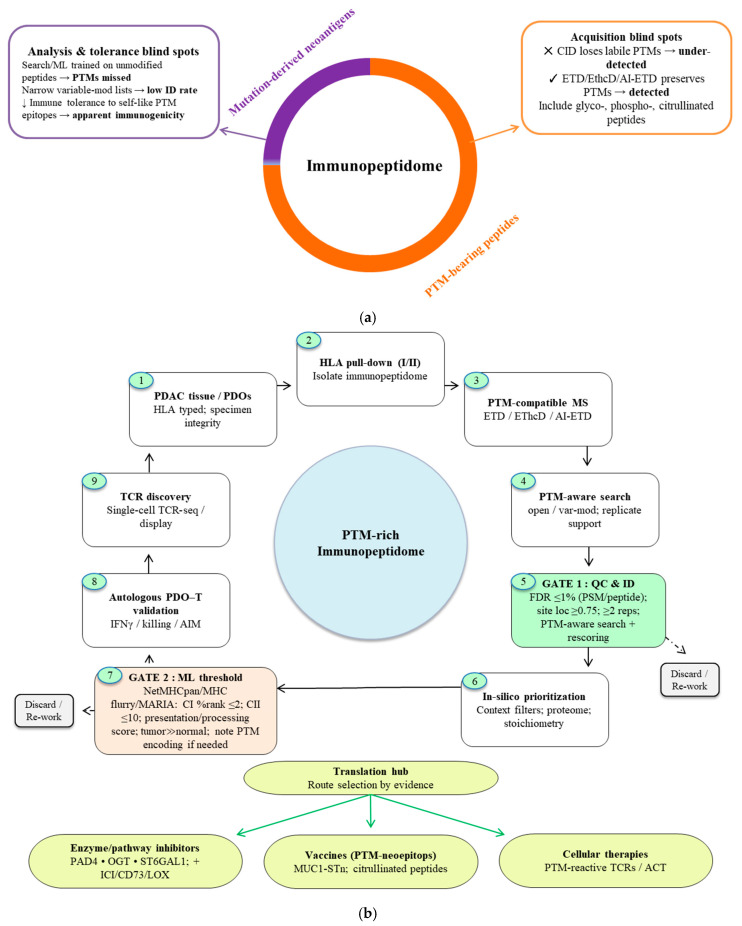
Decoding antigenic dark matter in PDAC. (**a**) Composition and detectability of the PDAC immunopeptidome. PTM-bearing peptides (orange) constitute the predominant fraction, whereas mutation-derived neoantigens represent a smaller subset (purple). This PTM-rich space is systematically under-recovered by (i) acquisition methods that strip labile groups (CID), (ii) analysis biases (search/ML models trained on unmodified peptides; restricted variable-mod settings), and (iii) immunological tolerance to self-like PTM epitopes together yielding the illusion of low immunogenicity. Electron-based fragmentation (ETD, EThcD, AI-ETD) preserves fragile modifications and improves detection and site localization. (**b**) Gated decoding pipeline from discovery to translation. PDAC tissue/PDOs → HLA pulldown → PTM-compatible MS → PTM-aware search → **Gate 1** applies standard QC (≤ 1% FDR, site localization ≥ 0.75, replicate support, PTM-aware search/rescoring) → **Gate 2** retains peptides meeting HLA-binding rank thresholds (Class I ≤ 2%, Class II ≤ 10%), favorable presentation/processing scores, and tumor-restricted expression, with surrogate PTM encodings documented where predictors lack native support. Candidates passing both gates are routed via a Translation Hub to (i) enzyme/pathway inhibitors (PAD4, OGT, ST6GAL1; often combined with ICI/CD73/LOX), (ii) PTM-neoepitope vaccines (e.g., MUC1-STn, citrullinated peptides), or (iii) cellular therapies (PTM-reactive TCRs/ACT).

Despite their immunological importance, PTM-derived antigens have been difficult to access because of several practical hurdles:

- Detection Limitations: Conventional mass spectrometry often fails to capture PTM-modified peptides owing to low abundance and chemical lability [83,84]. Advances in MS-based glycoproteomics including size-exclusion-based enrichment are improving the detection of sialylated epitopes that are particularly relevant in PDAC, including within the stromal compartment [85,86].
- Functional Validation: Establishing bona fide immunogenicity requires stringent in vitro and in vivo testing. CRISPR-engineered PDAC organoids, including constructs that recapitulate specific PTMs (e.g., p53 O-GlcNAcylation), are being leveraged to probe T cell reactivity and antigen processing [87,88].
- Clinical Proof-of-Concept: Early clinical experience with vaccines targeting citrullinated vimentin in PDAC, together with preclinical studies of PTM-focused vaccines, points to therapeutic promise. PTMs such as citrullination can yield tumor-associated antigens capable of driving immune responses [34,77,89]. A cohesive strategy to decode this “dark matter” of the immunopeptidome from PTM-compatible discovery platforms to the prioritization and translation of targetable epitopes is therefore essential (Figure 4).Collectively, these data position PTMs as a critical yet underutilized dimension of cancer immunotherapy. Systematically harnessing this antigenic “dark matter” may provide new strategies to overcome immune resistance in antigenically cold tumors such as PDAC.

Numerous studies indicate that PTM-modified HLA peptides are a reliable and detectable element of the immunopeptidome, with their prevalence significantly influenced by sample type, PTM class, and search approach [90,91]. Extensive re-analyses and targeted methodological publications illustrate a significant quantity of phosphorylated and glycosylated HLA ligands, highlighting that open/PTM-aware searches significantly enhance modified peptide identification, but PDAC-specific proportions remain indeterminate. These data together validate PTM-aware discovery in PDAC without excessive extrapolation of numerical fractions.

## 3. PTMs in Cancer Immunology: Mechanisms and Consequences

### 3.1. Modifying the Antigenic Code: The Role of PTMs in Epitope Diversification

PTMs operate as molecular switches that remodel the tumor immunopeptidome in real time, generating antigenic heterogeneity that exceeds the limits of the static genome [34,92]. In PDAC, three PTM classes glycosylation, phosphorylation, and citrullination emerge as dominant regulators of immune visibility, shaping antigen processing, presentation, and recognition in both immunostimulatory and immunosuppressive directions [34]. PDAC cells exhibit increased terminal sialylation (e.g., on MUC1/MUC16), which activates inhibitory Siglec-7/-9 on myeloid cells, resulting in antigen-presentation deficiencies and T cell suppression. Additionally, CAFs provide sialylated ligands, broadening this checkpoint beyond tumor cells [56]. Stabilizing N-glycans on PD-L1 extends surface residency and inhibits cytotoxic T cell activity, establishing a direct correlation between glycosylation and checkpoint efficacy [93]. These glyco-circuits, which come from tumors and stroma, are now known in PDAC as immune-evasion nodes that can be worked with [94].

### 3.2. Regulation of PTM Landscapes by Microenvironmental Factors

The spatial heterogeneity of the PDAC microenvironment imposes strong region-specific pressures on PTM patterning and its immunologic consequences [95]. Spatially resolved proteomics delineate discrete PTM gradients within tumors:

- Hypoxic cores show the enrichment of O-GlcNAcylated proteins including HIF-1α which perturbs peptide loading and complex stability on MHC-I, thereby diminishing antigen presentation [87,96].
- Stromal interfaces harbor abundant citrullinated extracellular matrix proteins that promote neutrophil extracellular trap (NET) formation; these structures foster immune evasion and facilitate metastatic dissemination through NET-associated immunomodulation [97,98].

### 3.3. PTM Interference and Immune Regulation

PTMs integrate metabolic, epigenetic, and stromal cues into coordinated immune-evasion programs. In PDAC, these interconnected circuits create context-dependent vulnerabilities that enhance tumor adaptability [36]. Three principal axes link extrinsic stress signals to immune regulation [99].

### 3.4. Metabolic Hypoxic Axis: Interplay Between O-GlcNAcylation and Phosphorylation

Under hypoxia, HIF-1α upregulates the hexosamine biosynthetic pathway, elevating UDP-GlcNAc and driving O-GlcNAcylation of key signaling proteins [87,100]. In PDAC, O-GlcNAcylation of β-catenin at serine 552 blocks GSK3β-mediated phosphorylation, preventing proteasomal degradation [101]. The resulting stabilization enhances β-catenin nuclear accumulation and transcriptional output, promoting metastatic behavior [87,101]. Concurrently, O-GlcNAcylation of pyruvate kinase M2 (PKM2) at threonine 405 suppresses its kinase activity, redirecting glucose flux toward glycosylation precursors and further reinforcing O-GlcNAcylation [102]. O-GlcNAcylation of β-catenin may block GSK3β-mediated phosphorylation, leading to β-catenin stabilization. This, in turn, could increase its presentation as an MHC-I epitope, potentially influencing immune recognition.

Hypoxia and metabolic rewiring (GFPT1/2 flux) enhance O-GlcNAc cycling in PDAC, altering signaling and antigen-processing proteins, which, therefore, diminishes effective MHC-I visibility in immune-excluded niches [103]. Recent PDAC results reveal an HBP–O-GlcNAc–YBX1 axis that alters cytokine production (e.g., IL-18) and the tumor immunological milieu, highlighting O-GlcNAcylation as a context-dependent immune modulator [59]. O-GlcNAc integrates hypoxia metabolism with immune editing in PDAC [104].

### 3.5. Epigenetic Immunogenic Axis: Circuits of Acetylation and Ubiquitination

Epigenetic control intersects with PTM-dependent antigen presentation. Histone deacetylase inhibitors (e.g., panobinostat) induce hyperacetylation of HSP90 at lysine 294 [105,106], which flags the chaperone for CHIP-mediated ubiquitination, proteasomal turnover, and exposure of otherwise cryptic client oncoproteins such as HER2/neu as immunogenic targets [107]. PDAC cells counter that by overexpressing the glycosyltransferase ST6GAL1, increasing PD-L1 sialylation, enhancing engagement of Siglec-9 on T cells, and dampening activation [57]. This layered resistance where epigenetic inputs drive PTM-based modulation of immune checkpoints illustrates a broader paradigm of PTM-enabled immune escape in PDAC [36].

### 3.6. Stromal Tumor Axis: Networks of SUMOylation and Citrullination

Within PDAC’s desmoplastic stroma, CAFs orchestrate immunosuppression through coordinated PTM signaling [108]. The activation of STAT3 shaped by microenvironmental cues including hypoxia can downregulate components of the MHC-I antigen-processing machinery (e.g., TAP1/TAP2), thereby impairing antigen display; activated SUMOylation can limit MHC-I antigen presentation and reduce tumor immunogenicity; preliminary preclinical studies on PDAC with SUMO-pathway blockage indicate the activation of antitumor immunity [109,110,111]. At the matrix interface, PAD4-driven citrullination of proteins such as fibronectin (e.g., arginine 38) promotes NET formation, fueling metastasis and establishing immunosuppressive niches [34,97]. The genetic inhibition of PAD4 in the KPC mouse model markedly reduces NETs and constrains metastatic progression, underscoring the functional importance of stromal tumor PTM crosstalk [112,113]. The creation of PAD4-dependent NETs leads to stromal entrapment, vascular problems, and T cell exclusion in PDAC. It also makes citrullinated self-peptides that can act as neoepitopes [98]. Targeting PAD4/NETs serves a dual purpose: it breaks down physical and chemical barriers to infiltration and reveals citrullinated antigens for vaccination or T cell treatments [98,114].

### 3.7. Evolutionary Consequences of PTM Interactions

Single-cell analyses of PTM states indicate that immunologic pressure can shape clonal selection according to specific modification patterns, evidencing adaptive evolution in malignant populations [115].

- Hybrid glyco-phospho-signaling on mucins: In PDAC, atypical glycosylation and phosphorylation of MUC1 are recurrent and contribute to immune escape. Hybrid glyco-phospho-PTMs on mucins remodel molecular conformation and binding interfaces, creating steric barriers to immune recognition and altering antigen processing and presentation [116,117].
- Enzymatic crosstalk among PAD4 and OGT: Tumors with altered PAD4 activity including putative loss-of-function variants frequently display concomitant O-GlcNAc transferase (OGT) overexpression, revealing coordinated dysregulation across PTM enzymes. The resulting imbalance between citrullination (PAD4) and O-GlcNAcylation (OGT) fosters a protumor immunosuppressive niche across multiple cancer types [58,118].

### 3.8. Immune Editing Pressures and Post-Translational Modification-Driven Antigenic Landscapes

PTMs provide a rapid, reversible layer of antigenic plasticity that operates independently of DNA sequence change, enabling tumors to recalibrate their immune visibility under the selective pressures of immune editing [76]. Unlike mutation-fixed neoantigens, PTM programs are enzymatically tunable, conferring swift immuno-adaptation and a survival advantage in hostile microenvironments [35].

### 3.9. Immune Evasion Induced by Hypoxia

In PDAC, hypoxia reprograms cellular metabolism and elevates O-GlcNAcylation. O-GlcNAcylation of β-catenin stabilizes the protein often by antagonizing phosphorylation, leading to its accumulation, activation of Wnt pathway transcriptional outputs, and promotion of proliferation and metastatic competence [101]. These post-translational changes also reshape the antigenic display of β-catenin, with downstream effects on antigen presentation and T cell recognition [119]. Moreover, changes in β-catenin after translation, such as O-GlcNAcylation, can affect its expression as a tumor antigen. Deviant β-catenin signaling is recognized for its role in immune evasion mechanisms in cancer, encompassing effects on antigen presentation and T cell identification [87,120]. Comprehensive investigations of PTMs and glycoproteomics in pancreatic cancer have elucidated their significant roles in tumor advancement and immunological regulation. Integrative PTM and glycoproteomic studies in pancreatic cancer link such β-catenin modifications to weakened antitumor immunity, including reduced CD8^+^ T cell recognition; these effects may be accentuated in individuals whose HLA alleles present β-catenin-derived epitopes [121,122].

### 3.10. Thymic Tolerance and Context-Dependent Immunogenicity

Citrullination mediated by PAD4 can generate novel self-peptides, some of which become MHC I-restricted neoepitopes. Central tolerance in the thymus typically purges high-affinity T cell clones against self, including many PTM-modified self-antigens, thereby constraining baseline immunogenicity [34,123]. Under inflammatory stress such as that induced by cytotoxic therapy, these tolerance barriers may be relaxed [124,125]. In PDAC, regimens like FOLFIRINOX can modulate immunity and increase tumor cell death, potentially unveiling previously concealed or newly modified epitopes [126]. Therapy- or disease-associated tissue damage further remodels the proteome via PTMs (e.g., citrullination), enabling the presentation of PTM-derived peptides by MHC molecules and, in defined contexts, reshaping T cell responses [34,127,128]. Functional data confirms the existence of PTM-reactive T cells in PDAC, including T cells targeting citrullinated enolase and vimentin in patients, alongside glycopeptide vaccines that elicit MUC1-specific T cell responses in models [50,129].

## 4. The PTM Landscape in Pancreatic Cancer

### 4.1. The PDAC Paradox: Minimal Mutational Load, Elevated Antigenic Complexity

PDAC presents a striking immunological paradox: it exhibits an exceptionally low TMB (often <1 nonsynonymous mutation per megabase) that is, typically associated with poor responsiveness to checkpoint blockade [130]; yet demonstrates pronounced antigenic complexity largely sculpted by diverse PTMs [36,131]. This discrepancy challenges neoantigen-centric models of tumor immunogenicity and underscores PTMs as key determinants of the PDAC immune milieu [132,133].

### 4.2. Universal Hyperactivation of PTM-Regulating Enzymes

A non-mutational hallmark of PDAC is the widespread hyperactivation of PTM-controlling enzymes, driven by convergent oncogenic and microenvironmental inputs [134]. Mutant KRAS, via MAPK MYC signaling, upregulates multiple PTM enzymes including the glycosyltransferase ST6GAL1, thereby reprogramming metabolism and signaling independently of genomic change [57,135].

Severe hypoxia engages the hexosamine biosynthetic pathway (HBP), increasing O-GlcNAcylation and fueling OGT activity; this in turn stabilizes HIF-1α and sustains hypoxic signaling [100,136]. Although direct evidence for PAD4 and ST6GAL1 as explicit downstream effectors within this HIF-1α axis is limited, they remain as plausible targets. A self-reinforcing circuit marked by OGT-mediated O-GlcNAcylation and tumor hypersialylation through ST3GALs drives proteome-wide remodeling and immune evasion, enhancing PD-L1 stability and promoting Siglec-mediated polarization of tumor-associated macrophages (TAMs) [58,94]. While PTM-driven, non-mutational neoantigen generation highlights these enzymes as attractive immunotherapeutic targets, direct evidence specifically implicating PAD4 in this loop is still lacking [137].

### 4.3. Spatial Heterogeneity of Post-Translational Modifications in the Tumor Microenvironment

Spatial profiling reveals a regionally organized PDAC microenvironment with discrete immune neighborhoods and tertiary lymphoid-like structures, suggesting localized immunologic niches. Although PTMs are likely regulated within this architecture, patterned distributions remain to be directly demonstrated [138,139].

Spatial proteomics identifies hypoxic zones in PDAC, and hypoxic cores can downregulate MHC-I presentation through HIF-1α–dependent and autophagy-mediated mechanisms [96,140]. However, direct accumulation of O-GlcNAc-modified HIF-1α or phospho-metabolic enzymes in these regions has yet to be shown [141]. PAD4 activity promotes histone citrullination and NET formation phenomena linked to immune exclusion and progression—yet stromal ECM-specific citrullination and its creation of physical barriers to T cell entry remain to be established [142,143,144]. CAFs in PDAC display enriched sialylation; sialic acids on CAFs can engage Siglec-7/-9/-10 on immune cells to enforce immunosuppression. While spatial omics has mapped immune niches and stromal architecture, direct visualization of sialylated mucin gradients at tumor–stroma borders still needs confirmation [141,145,146,147]. Collectively, these observations indicate that PDAC immune escape is spatially programmed by compartmentalized PTM networks, now accessible only through advanced spatial omics.

### 4.4. Stromal Contributions and Tumor Microenvironmental Influences

The desmoplastic stroma, particularly CAFs, actively reshapes the PTM landscape through metabolic and signaling crosstalk, including CAF-derived exosomes [148,149]. Although ST6GAL1-mediated α-2,6 sialylation is increased in PDAC and generates immunosuppressive glyco-ligands that can engage inhibitory Siglecs, a direct link from CAF exosomal miR-155 to ST6GAL1 upregulation remains to be shown [57,150]. In parallel, CAF-derived TGF-β suppresses antigen-processing genes (e.g., TAP2, ERAP1, β2-microglobulin) via Smad3, reducing MHC-I surface expression and dampening tumor immunogenicity [151]. Activation of SUMOylation pathways has likewise been implicated in restricting MHC-I presentation, raising the possibility of TGF-β–SUMOylation crosstalk in PDAC immune evasion, though direct SUMOylation of TAP/LMP components has not been demonstrated [111,152]. PTMs directly influence diagnosis. The CA19-9 biomarker is a sialylated glycoform, with its levels and detectability regulated by tumor-specific glycosylation mechanisms. Certain glycoforms exhibit superior diagnostic capabilities compared to others [39,85].

### 4.5. Case Studies and Notable PTM-Modified Antigens in PDAC

Glycosylated MUC1: Truncated O-glycans (Tn, STn) create a bulky glycocalyx that sterically shields peptide epitopes from CD8^+^ T cell surveillance [153]. The sialylated STn variant also binds inhibitory Siglec-7/-9 on NK and CD8^+^ T cells, promoting functional exhaustion and helping explain the limited efficacy of MUC1-targeted vaccines [154,155,156].

Phosphorylated p53: Beyond mutation-derived neoantigens, phospho-p53 (e.g., Ser392) yields MHC-I-presented phosphopeptides that broaden targetable epitopes [157,158]. Although phosphopeptide presentation is documented in several cancers, context-specific effects such as Treg recruitment by these epitopes remain hypothetical and unproven in PDAC [159,160].

Annexin A2 / CXCL1: Annexin A2 supports EMT, invasion, and metastasis, but roles for its S-nitrosylation under hypoxia- or iNOS-driven stress are not yet characterized. Separately, CXCL1 promotes MDSC expansion and recruitment, fostering immune-excluded niches [161].

Together, these exemplars illustrate how discrete PTMs can rewire antigen visibility and immune outcomes, reinforcing the need for PTM-aware antigen discovery and therapeutic design in PDAC.

## 5. Challenges: Why PTMs Remain Dark Matter in Oncology

### 5.1. Challenges in Technical Detection

Deciphering the PTM-bearing immunopeptidome remains technically difficult because standard mass spectrometry workflows are poorly suited to capture these species [162,163,164]. Modified peptides typically occur at low stoichiometry, ionize inefficiently, and frequently undergo neutral loss during fragmentation [165,166]. As a result, labile modifications such as O-GlcNAcylation and citrullination are often cleaved under collision-induced dissociation (CID), yielding underrepresentation or misassignment [167,168]. The wide dynamic range of the proteome further obscures rare PTM peptides beneath abundant unmodified counterparts [169]. Although enrichment strategies (e.g., lectin affinity for glycopeptides; antibody-based capture such as PTMScan) improve recovery, they introduce method-specific biases and remain limited for uncommon or emergent PTMs [170,171]. Compounding this, conventional immunopeptidomics pipelines are optimized for unmodified ligands, systematically under-detecting PTM-derived antigens [172,173,174]. Together, these challenges create a persistent analytical blind spot that conceals much of the antigenic dark matter in PDAC.

Table 3 contrasts traditional CID/HCD with PTM-compatible electron-based processes (ETD/EThcD) and the newer AI-ETD to show how real trade-offs can be measured.

### 5.2. Complexities of HLA Presentation and Immune Recognition

A central barrier to harnessing PTM neoepitopes is that current HLA-binding predictors trained largely on unmodified peptides poorly capture how modifications alter peptide–MHC stability: a given PTM may disrupt anchor contacts, be neutral, or enhance binding to generate strong neoepitopes [181,182,183]. While central tolerance typically deletes T cells with high affinity for self, accumulating evidence indicates that T cells specific for PTM-derived epitopes (e.g., phosphorylation-induced targets) may escape thymic deletion; however, such clones often exhibit low functional avidity, limiting cytotoxic efficacy [173]. PTMs can also reconfigure TCR recognition geometry phosphate or glycan moieties may become dominant contact features in the peptide–MHC–TCR interface, shifting signaling outcomes from activation to anergy or tolerance [157,184,185,186]. These constraints underscore the need to prioritize PTMs that are tumor-restricted or abundantly presented to ensure sufficient T cell engagement despite tolerance.

### 5.3. Telling the Difference Between Driver and Passenger PTMs

Not every PTM in PDAC has a functional meaning. We propose an evidence-weighted checklist to focus on probable drivers rather than incidental passengers: (i) Specificity and perturbability of the enzyme as a writer or eraser of PTMs (e.g., OGT, ST6GAL1, PAD4, kinases), where genetic or pharmacologic modulation can alter the PTM and influence the downstream immune phenotype (e.g., OGT, ST6GAL1, PAD4, kinases) whose genetic or pharmacologic modulation shifts the PTM and the downstream immune phenotype (Table 2 entries and enzyme sections); (ii) tumor-specific enrichment versus matched normal across independent cohorts, ideally with measurable stoichiometry or dynamic changes along hypoxic or metabolic gradients [94,135,136,147]; (iii) spatial co-localization with immune components (e.g., Siglec+ myeloid cells, Tregs, PD-L1high niches) shown by spatial PTMs that fulfill many criteria, including enzyme-linked causality [145], tumor-restricted enrichment, spatial immune coupling, and CRISPR/inhibitor validation, should be prioritized as key drivers and progressed towards vaccines, TCR treatments, or enzyme-targeted therapeutics.

## 6. Novel Solutions and Innovations

New analytical and functional platforms are beginning to illuminate PTM-derived epitopes. Hybrid fragmentation strategies that combine electron transfer with higher-energy collision dissociation (EThcD) better preserve fragile modifications (e.g., O-glycans) and enable confident site localization [168,187]. Electron-based methods such as ETD and AI-ETD similarly minimize PTM loss, improving the identification of phosphosites and citrullinated residues [188,189,190]. On the computational side, machine-learning models trained on PTM-enriched immunopeptidome datasets are improving HLA-binding and presentation predictions for modified peptides, outperforming models built solely on unmodified sequences [191,192,193,194]. Incorporating these additional layers of information may enhance models’ predictive accuracy for PTM-driven immune targets. This indicates that they are not solely dependent on peptide sequences, hence enhancing the specificity of predictions for therapeutic applications. Recent advancements in deep learning have shown that including local sequence context and post-translational modification-specific characteristics significantly enhances forecast accuracy [195]. For functional validation, patient-derived organoid T cell co-cultures provide autologous, HLA-matched systems to test PTM neoantigen immunogenicity under physiologic constraints [196,197]. In parallel, high-throughput TCR discovery pipelines are uncovering rare PTM-reactive receptors from tumor-infiltrating lymphocytes or donor repertoires, supporting the development of engineered TCR therapies [196,197,198,199,200]. Together, these advances outline a practical path to render PTM biology actionable in oncology.

## 7. Therapeutic Horizons: Targeting PTM Antigens

To prevent mixing up maturity levels, we categorized PTM-anchored therapies into three groups: tested in humans, promising preclinical, and conceptual/early discovery (Table 4).

### 7.1. Vaccines Aimed at PTM Neoepitopes

Vaccines targeting PTM-derived epitopes (notably aberrant glycosylation) offer a rational path to overcome tolerance and enhance antitumour immunity in PDAC [208,209]. The principal obstacle is that many PTM-modified epitopes originate from self-proteins, invoking B and T cell tolerance mechanisms [210]. Nonetheless, PTM-targeted vaccines have the potential to overcome these barriers and strengthen tumor-specific immunity in PDAC [211,212]. Second-generation synthetic glycopeptide platforms such as MUC1-STn constructs linked to T-helper epitopes or formulated on nanoparticle carriers have elicited class-switched IgG and cytolytic T cell responses that recognize glycosylated MUC1 on tumor cells [50,211,212,213,214]. In parallel, vaccines directed at citrullinated antigens (e.g., vimentin, α-enolase) have advanced into early-phase clinical or preclinical testing; by engaging HLA class II, these constructs can circumvent central tolerance and induce robust CD4^+^ and, in some contexts, CD8^+^ responses in poorly immunogenic settings [74,129,215,216,217]. Although the dominant signal is often Th1-biased CD4^+^ immunity, efficacy in low-immunogenicity tumors indicates potential for breaking tolerance and supporting CD8^+^ activation [129]. Importantly, PTM-focused vaccines appear most effective when combined with checkpoint blockade: preclinical studies show that responses to PTM-derived epitopes depend on relief from PD-1/CTLA-4-mediated suppression to maintain durable antitumor activity [218,219,220,221]. Collectively, these data chart a transition from proof-of-concept glycopeptide vaccines toward a new generation of PTM-informed immunization strategies that broaden PDAC’s targetable antigenic space. If PTM-based vaccines disrupt immunological tolerance, the resulting T cells may rapidly become fatigued within the tumor microenvironment (TME), which inhibits the immune system. This highlights the importance of combining these vaccinations with immune checkpoint inhibitors (ICIs) to prevent T cell exhaustion and enhance sustained antitumor responses [222].

### 7.2. Enzyme Inhibitors to Block PTM Machinery

An alternative to antigen-directed vaccination is to pharmacologically disrupt the enzymatic programs that generate immunosuppressive PTM signatures. In PDAC models, PAD4 inhibition (e.g., GSK484) reduces NET formation and curtails tumor growth [113,223,224], while PAD4 deletion or treatment with JBI-589 impairs metastasis and markedly improves responses to immune checkpoint blockade [113,225]. Key transcriptional regulators, such as β-catenin, are affected by OGT inhibition, which prevents hyper-O-GlcNAcylation, while OGT inhibitors restore cGAS–STING-dependent antigen presentation and elicit potent CD8+ T cell-mediated antitumor immunity [58,226]. Inhibiting tumor-associated sialyltransferases such as ST6GAL1 reduces hypersialylation, disrupts Siglec–sialoglycan interactions that dampen effector function, and enhances NK and T cell cytotoxicity [155,227,228,229,230,231]. Finally, stromal-targeted approaches such as lysyl oxidase (LOX) inhibition can soften the ECM, improve immune infiltration, and complement PTM-directed strategies [232,233,234]. Together, inhibitors of PTM enzymes constitute a class of metabolic immunotherapeutic agents capable of reprogramming PDAC from an immune-excluded state to an immune-responsive state.

### 7.3. Risks of Clinical Translation

Alongside these opportunities, PTM-targeted approaches come with clear safety trade-offs. Hitting broadly expressed epitopes like citrullinated peptides can break tolerance and cause autoimmunity [235]. This risk may go up when ICIs are added [236]. Enzyme-directed medicines (e.g., OGT or PAD4 inhibitors) can also have effects on-target and off-tumor because these enzymes help normal tissue work [100,237]. To mitigate these risks, candidates must demonstrate significant tumor restriction, validated HLA presentation, and T cell specificity in autologous systems. They should also participate in stepwise, closely monitored trials (with careful dose escalation, predefined stopping rules, and serial assessments of autoantibodies, cytokines, and organ function). To protect important physiology, context-dependent or localized techniques including targeted administration, transient/inducible regimens, and selective enzyme inhibition (isoform/complex-specific) should be used whenever possible.

## 8. Outlook and Strategic Recommendations

### 8.1. Thorough Mapping of PTM–Immune Interfaces

Targeting the stroma offers a complementary route to unlock antitumor immunity in PDAC. Inhibiting lysyl oxidase (LOX) can lessen collagen crosslinking and extracellular matrix (ECM) stiffening hallmarks of PDAC, thereby improving immune cell ingress [233,238,239,240]. Although direct PDAC evidence remains limited, studies on other solid tumors show that LOX blockade reduces matrix rigidity, facilitates CD8^+^ T cell trafficking, and augments responses to anti–PD-1 therapy [241,242]. In parallel, advances in deep immunopeptidomics such as applying EThcD or AI-ETD fragmentation to HLA pulldown samples increase recovery and confident localization of labile PTM-modified peptides that commonly escape conventional workflows [180,243,244]. Critically, machine-learning models trained on PTM-enriched immunopeptidome datasets are beginning to improve the prediction of PTM–HLA interactions. Together, these developments motivate the creation of a PTM antigen atlas analogous to TCGA or CPTAC in proteogenomics to convert disparate PTM observations into a standardized resource for therapeutic target triage.

### 8.2. Immunogenicity Assessment in Patient-Derived Organoids and Models

A definitive evaluation of PTM neoantigen function requires models that preserve human tumor context. Patient-derived organoids (PDOs) co-cultured with autologous peripheral or tumor-infiltrating immune cells provide an HLA-matched platform to measure antigen-specific T cell priming, effector function, and cytotoxicity under physiologically relevant conditions [197,245,246]. These immuno-organoid systems can be precisely edited with CRISPR/Cas9 to delete key PTM enzymes (e.g., PAD4, OGT) or to introduce defined PTM neoantigen constructs, enabling causal tests of how specific modifications alter immune recognition [247,248]. Because PDOs retain each patient’s native HLA background and, thus, their history of thymic tolerance, they also help prioritize modifications with genuine translational potential [196,248]. When combined with high-throughput TCR discovery pipelines, this framework links antigen identification to epitope prioritization, ensuring that only actionable PTM targets advance.

### 8.3. Collaborative Frameworks: International PTM Antigen Discovery Consortia

Systematically charting the PTM “dark matter” in cancer immunology will require coordinated, multi-institutional efforts modeled on initiatives such as the Human Immuno-Peptidome Project (HIPP). An international PTM Antigen Discovery Consortium could harmonize PTM-focused immunopeptidomics protocols, build centralized repositories of PTM–HLA ligands, and enable the sharing of scarce patient samples and specialized reagent libraries [249,250]. Such a structure would reduce duplication, enable cross-cohort validation, and generate a robust reference atlas of PTM epitopes. By integrating immunopeptidomics with spatial proteomics and clinical HLA typing, these networks could stratify patients by HLA allotype and PTM phenotype, informing the rational design of off-the-shelf vaccines and adoptive cell therapies [251,252]. Establishing this collaborative infrastructure is essential to move PTM antigens from isolated findings to clinically actionable immunotherapy targets.

## 9. Additional Regulatory Mechanisms in Pancreatic Cancer

In PDAC, the SUMO conjugation machinery is often overexpressed, which leads to better transcriptional regulation, faster DNA repair, continued cell-cycle progression, and more resistance to therapy, especially in aggressive tumor phenotypes [253]. Pharmacologic inhibition of this system using TAK-981 impairs normal mitotic processes, resulting in G2/M arrest in PDAC cells and significantly inhibiting tumor growth in animal models by simultaneously enhancing antitumor immunity [254]. Additionally, oncogenic KRAS has been demonstrated to facilitate SUMOylation-dependent release of extracellular vesicles and promote lymphangiogenesis, hence enhancing the interplay between intracellular oncogenic signaling and the tumor microenvironment [255]. More recent findings reveal that SUMOylation also promotes immune evasion in PDAC by modulating pathways such as TIGIT/CD155, underlining potential intersections with immunotherapeutic methods [256].

β-Hydroxybutyrylation: The ketone body β-hydroxybutyrate (β-HB) can trigger histone β-hydroxybutyrylation, a change that boosts the transcription of antioxidant genes and protects pancreatic cells from oxidative stress linked to ferroptosis [257]. In malignant contexts, β-HB has been demonstrated to facilitate ferroptotic cell death by inhibiting caveolin-1 (CAV1) production, hence illustrating its dual regulatory role in pancreatic disease [258].

Methylation: In PDAC, many protein methyltransferases, such as CARM1 and PRMT1, are crucial in regulating cellular metabolism and gene expression [259]. Dysregulation of histone methylation, especially via hyperactive EZH2 and G9a enzymes, facilitates chromatin remodeling that enhances tumor aggressiveness and is associated with poor patient outcomes [260].

Ferroptosis: Lipid peroxidation-induced ferroptosis represents a unique susceptibility in pancreatic cancer cells [261]. The glutathione peroxidase GPX4, the exchange of cystine and glutamate, and the balance of iron inside the cell all tightly control this process. Inducing ferroptosis impairs redox homeostasis, inhibits tumor proliferation, and increases the sensitivity of resistant pancreatic ductal adenocarcinoma (PDAC) to current therapy strategies [262].

## 10. Conclusions

PTMs represent a dominant yet incompletely charted dimension of antigenicity in PDAC. Spatially organized modification programs O-GlcNAcylation and citrullination enriched in hypoxic cores, heightened sialylation at tumor–stroma interfaces, and context-dependent phosphorylation at the periphery converge to enforce immune exclusion by suppressing MHC-I display, stabilizing PD-L1, and fostering NET-mediated physical barriers. Overcoming this immune refractoriness calls for a PTM-aware, end-to-end pipeline: (i) preserve labile PTMs during HLA-peptidomics using ETD/EThcD/AI-ETD; (ii) analyze spectra with machine-learning models trained on PTM-enriched datasets; (iii) functionally triage candidates in autologous PD-T-cell co-cultures; and (iv) translate prioritized targets into PTM-directed vaccines and enzyme inhibitors (e.g., OGT, PAD4, ST6GAL1), coupled with stromal remodeling where indicated. By prioritizing PDAC-relevant PTM immune axes and standardizing PTM-centric workflows, the field can enable robust target discovery and rational combinations with checkpoint blockade moving PTM antigens from “dark matter” to a tractable therapeutic class.

## Figures and Tables

**Table 1:** Clinical failures of mutation-targeted immunotherapies in PDAC.

Therapy/Agent	Target Mutation(s)	Trial Phase/ID	ORR/Clinical Outcome	Resistance Mechanism (TME)	Resistance Mechanism (Immune)	Key Insights	Ref
**GI-4000 (yeast vaccine)**	Mutant KRAS	Phase II (NCT00300950)	No survival benefit; immune responses in subset	Desmoplastic stroma; immune exclusion	Weak effector T cell priming despite immune activation	Residual tumor drives immune response. Immune activation correlates with survival. Proteomic biomarkers predict response.	[26]
**mRNA-5671 (V941)**	KRAS G12D/V/R/C	Phase I (NCT03948763)	Trial terminated (futility)	CAF/MDSC-mediated immune suppression	Lack of durable T cell responses to KRAS epitopes	Personalized vaccine approach. Combination therapy potential. Safety and tolerability.	[27]
**ELI-002 (liposomal vaccine)**	KRAS G12D/R	Phase I (NCT04853017)	~21% KRAS-specific T cell response	Antigen presentation barriers; TME-driven T cell exhaustion	Limited cytotoxicity of expanded CD8^+^ T cells	Personalized approach Combination potential Preliminary immune response	[28]
**TP53 synthetic long-peptide vaccine**	Mutant TP53	Phase I/II	0% ORR in PDAC cohort	Stromal desmoplasia; TGF-β signaling	Immune ignorance; Treg/MDSC induction	TP53 mutation as a prognostic marker. Immune landscape correlation. Therapeutic implications.	[29]
**Durvalumab ± Tremelimumab (ICIs)**	PD-1 / CTLA-4	Phase II (NCT02558894)	3.1% (Durva), 0% (combo)	Low neoantigen load; CD73/CD39 adenosine pathway	T cell exclusion; exhausted phenotype	Limited efficacy of dual immune checkpoint inhibition. Absence of response to monotherapy. safety profile.	[17]
**Pembrolizumab (ICI)**	PD-1	Phase II (KEYNOTE-158, NCT02628067)	~1% ORR in PDAC, benefit restricted to MSI-H subset	Poor T cell infiltration; immunosuppressive stroma	Lack of neoantigens in MSS tumors; PD-1^+^ exhausted T cells	Efficacy of pembrolizumab in MSI-H/dMMR cancers. Durable responses and survival outcomes. Safety profile consistent with previous experience.	[30]
**Personalized mRNA vaccines**	Patient-specific neoAg	Phase I (NCT04161755, etc.)	Limited efficacy in PDAC	Low mutational load limits high-affinity targets	Immunodominance of weak responses; MDSC suppression	High response rate. Long-term T cell persistence. Correlation with delayed recurrence. Safety profile.	[31]
**Sotorasib (KRAS G12C inhibitor)**	KRAS G12C (rare in PDAC)	Phase I/II (CodeBreaK100)	~21% ORR, short-lived	Very low G12C prevalence (~1–2%); adaptive resistance	Epitope loss; bypass pathway activation	Efficacy of Sotorasib in KRAS p.G12C-mutated pancreatic cancer. Objective response rate. Safety profile.	[32]
**TCR-T (engineered TCR, KRAS G12D)**	KRAS G12D (HLA-C*08:02)	Case report (NCT03745326)	Partial response (~72% shrinkage)	Tumor heterogeneity; stromal barrier	Restricted HLA subtype; limited persistence of engineered TCR clones	Targeted TCR gene therapy. Objective tumor regression. Immune response monitoring.	[33]

**Table 2:** PTM-mediated immune regulation in PDAC: mechanisms, biomarkers, and therapeutic opportunities.

PTM Type	Key Enzymes	Example Substrates/Sites	Immune Mechanism in PDAC (Concise)	Biomarker Potential	Therapeutic Strategies	Ref.
Aberrant O-glycosylation and hypersialylation (Tn/STn; α2-6 sialylation)	GALNTs, ST6GAL1, ST3GALs	MUC1/MUC16; PD-L1; EGFR	Siglec-7/9 engagement → myeloid immunosuppression; epitope masking	ST6GAL1 overexpression; hypersialylation signatures; MUC1-STn	Sialyltransferase inhibitors; anti-Siglec; desialylating biologics; glycopeptide vaccines	[56,57]
O-GlcNAcylation (HBP–OGT axis)	OGT, GFAT1/2	PD-L1; SIRT7; YBX1	PD-L1 stabilization; M2-skewing via GFPT2→O-GlcNAc→YBX1/IL-18	Global O-GlcNAc; OGT/GFPT2 expression	OGT/HBP inhibitors; ± PD-1/PD-L1 blockade	[58,59]
Citrullination → NETs	PAD4, PAD2	Histone H3Cit; extracellular vimentin	NET-mediated T cell exclusion; pro-metastatic trapping	H3Cit (tissue/serum); PAD4 expression/activity	PAD4 inhibitors; DNase; NET-targeting ± ICB	[60]
Phosphorylation (MAPK/ERK; JAK/STAT)	EGFR→MEK/ERK; JAK/STAT3	p-ERK; p-STAT3	EGFR/MAPK↑ PD-L1 on tumor cells; ERK-i + anti-PD-L1 synergy	p-ERK/MEK; STAT3 activity	MEK/ERK or STAT3 inhibitors + ICB	[61,62]
SUMOylation	SAE1/UBA2 (E1), UBC9 (E2), PIAS E3s	Broad (MYC-linked programs)	SUMO-addicted MYC-high subtype; SUMOi activates antitumor immunity	SUMO-pathway signature; UBC9/SAE1 IHC	TAK-981 (sumoylation E1 inhibitor) ± ICB	[63]
Ubiquitination/Deubiquitination (PD-L1 stability)	TRIM E3s; USP8, CSN5 (DUBs)	PD-L1, β-catenin	DUB-stabilized PD-L1; DUB-i + anti-PD-L1 boosts CD8^+^ T cells	DUB (USP8/CSN5) expression; PD-L1 levels	DUB inhibitors (USP8); PD-L1 degraders/PROTACs; E3 activation	[64,65]
Acetylation (HDAC/p300-CBP)	HDACs; p300/CBP	Histones; NF-κB/STATs	APM/MHC-I repression; HDACi restores CTL sensitivity	HDAC expression; histone acetylation patterns	HDAC inhibitors ± ICB; epigenetic immunotherapy	[66]
S-Nitrosylation (iNOS/NO)	iNOS (NOS2)	STAT3, RAF1, multiple targets	Proteome-wide S-nitrosylation; myeloid NO suppresses T cells	iNOS expression; S-nitrosylome panels	iNOS inhibition; myeloid-targeting ± ICB	[67,68]

**Table 3:** PTM peptide fragmentation procedures.

Workflow	PTM Retention	Best for	Strengths	Limitations	Ref
CID/HCD	Low for labile PTMs (neutral loss common)	Fast general proteomics; glyco survey (oxonium ions)	High speed and IDs; robust DIA/DDA backbone	Neutral loss hampers site localization (e.g., phospho); O-GlcNAc/O-glyco often degrade	[175,176]
ETD	High (preserves labile PTMs)	Phospho, O-GlcNAc, fragile HLA peptides	Retains labile groups; improves site calls; complements HCD	Lower efficiency for short/low-charge peptides; slower	[177]
EThcD (ETD + gentle HCD)	High with better sequence coverage	O-glycopeptides; broader PTM mapping	Outperforms ETD/HCD for O-glyco; richer spectra (b/y + c/z)	Slightly slower; needs ETD-capable Orbitrap/Tribrid	[178]
AI-ETD (activated-ion ETD)	High with higher fragmentation yield	Phospho, intact glyco (incl. O-glyco); top/middle-down	More IDs and coverage than ETD; better for low-charge density precursors	Requires IR-enabled ETD cell; throughput < HCD	[179]
EThcD for HLA immunopeptidomes	Preserves fragile motifs; ↑ sequence coverage vs. HCD	HLA-I (9–12mers; internal Arg, low-charge density)	Deeper HLA coverage with minimal duty-cycle penalty (newer hardware)	Needs ETD/EThcD-capable instruments	[180]

**Table 4:** PTM-anchored immunotherapy approaches by translational stage.

Stage	Modality	Representative Example	Indication(s) Tested	Current Status	Ref
Tested in humans	Glycopeptide/peptide vaccine (MUC1)	MUC1 100-mer peptide + adjuvant (or DC-pulsed MUC1)	Pancreatic cancer (resected/advanced)	Phase I trials show safety and immunogenicity; no definitive efficacy	[201]
	Phosphopeptide vaccine	HLA-I phosphopeptides (e.g., pBCAR3, pIRS2)	Melanoma/solid tumors (first-in-human)	Phase I: safe, immunogenic; supports further development	[202]
	Myeloid checkpoint blockade (anti-Siglec-15)	NC318 (± pembrolizumab)	Solid tumors (multitumor baskets)	Ongoing Phase I/II; mixed early activity	[203]
	SUMO-pathway inhibitor	Subasumstat/TAK-981	Solid and hematologic malignancies	Phase I/II: acceptable safety; early signals (combo settings)	[204]
	Citrullinated peptide vaccine	Modi-1 (cit-vimentin/enolase)	Solid tumors	Solid tumorsPhase I/II ongoing	[205]
Promising preclinical	Citrullinated peptide vaccines	Modi-1 (cit-vimentin/enolase)	Solid tumors (basket)	Phase I/II ongoing; preclinical efficacy + early clinical immunogenicity reported	[206]
	Global sialylation blockade	3Fax-Neu5Ac derivatives/prodrugs	Solid tumors (preclinical)	In vivo reduction in Siglec signaling; safety optimization ongoing	[207]

## Data Availability

There is no new information in this review article. All evidence that backs up the discussion and conclusions come from studies that have already been published and are cited in the manuscript.

## Abbreviations

The following abbreviations are used in this manuscript:

ACT	Adoptive Cell Therapy
APM	Antigen-Processing Machinery
β-HB	β-hydroxybutyrate
β2M	β2-microglobulin
CA19-9	Carbohydrate Antigen 19-9
CAF	Cancer-Associated Fibroblast
CARM1	Coactivator-Associated Arginine Methyltransferase 1
CAV1	Caveolin-1
CD	Cluster of Differentiation
CID	Collision-Induced Dissociation
CTLA-4	Cytotoxic T-Lymphocyte-Associated Protein 4
CXCL	C-X-C Motif Chemokine Ligand
DIA	Data-Independent Acquisition
DNA	Deoxyribonucleic Acid
DUB	Deubiquitinating Enzyme
ECM	Extracellular Matrix
EGFR	Epidermal Growth Factor Receptor
EMT	Epithelial–Mesenchymal Transition
ENO1	Enolase 1
ERAP1	Endoplasmic Reticulum Aminopeptidase 1
ERK	Extracellular Signal-Regulated Kinase
ETD	Electron Transfer Dissociation
EThcD	Electron Transfer/Higher-Energy Collision Dissociation
EZH2	Enhancer of Zeste Homolog 2
FDR	False Discovery Rate
FOXP3	Forkhead Box P3
FOLFIRINOX	Folinic Acid, Fluorouracil, Irinotecan, Oxaliplatin
GALNTs	Polypeptide N-Acetylgalactosaminyltransferases
GFAT	Glutamine:Fructose-6-Phosphate Aminotransferase
GPX4	Glutathione Peroxidase 4
GR	Glucocorticoid Receptor
GSK3β	Glycogen Synthase Kinase 3 Beta
HBP	Hexosamine Biosynthetic Pathway
HCD	Higher-Energy Collisional Dissociation
HDAC	Histone Deacetylase
HER2	Human Epidermal Growth Factor Receptor 2
HIF	Hypoxia-Inducible Factor
HIPP	Human Immuno-Peptidome Project
HLA	Human Leukocyte Antigen
HSP	Heat Shock Protein
ICI	Immune Checkpoint Inhibitor
IL	Interleukin
iNOS	Inducible Nitric Oxide Synthase
JAK	Janus Kinase
KRAS	Kirsten Rat Sarcoma Viral Oncogene Homolog
LOX	Lysyl Oxidase
MAPK	Mitogen-Activated Protein Kinase
MDSC	Myeloid-Derived Suppressor Cell
MHC	Major Histocompatibility Complex
MS	Mass Spectrometry
MSS	Microsatellite Stable
MSI-H	Microsatellite Instability-High
MUC	Mucin
NET	Neutrophil Extracellular Trap
NF-κB	Nuclear Factor Kappa B
NK	Natural Killer
NO	Nitric Oxide
NOS	Nitric Oxide Synthase
OGT	O-GlcNAc Transferase
ORR	Objective Response Rate
PAD	Peptidylarginine Deiminase
PD-1	Programmed Cell Death Protein 1
PD-L1	Programmed Death-Ligand 1
PDAC	Pancreatic Ductal Adenocarcinoma
PDO	Patient-Derived Organoid
PIAS	Protein Inhibitor of Activated STAT
PKM2	Pyruvate Kinase M2
PRMT	Protein Arginine Methyltransferase
PTM	Post-Translational Modification
QC	Quality Control
RAF1	Raf-1 Proto-Oncogene, Serine/Threonine Kinase
SER	Serine
STAT	Signal Transducer and Activator of Transcription
ST6GAL1	β-Galactoside α-2,6-Sialyltransferase 1
ST3GAL	β-Galactoside α-2,3-Sialyltransferase
STING	Stimulator of Interferon Genes
SUMO	Small Ubiquitin-like Modifier
TAM	Tumor-Associated Macrophage
TAP	Transporter Associated with Antigen Processing
TCR	T cell Receptor
TGF-β	Transforming Growth Factor Beta
Th	T Helper Cell
TIGIT	T Cell Immunoreceptor with Ig and ITIM Domains
TMB	Tumor Mutational Burden
TME	Tumor Microenvironment
Tn	Thomsen-nouvelle Antigen
TP53	Tumor Protein P53
Treg	Regulatory T Cell
TSA	Tumor-Specific Antigen
UBA2	Ubiquitin-Like Modifier Activating Enzyme 2
UBC9	Ubiquitin Conjugating Enzyme 9
UDP	Uridine Diphosphate
USP	Ubiquitin Specific Peptidase
